# GP trainees’ perceptions on learning EBM using conversations in the workplace: a video-stimulated interview study

**DOI:** 10.1186/s12909-020-02051-2

**Published:** 2020-05-06

**Authors:** Lisanne S. Welink, Esther de Groot, Peter Pype, Kaatje Van Roy, Iris D. van den Wittenboer, Marie-Louise E. L. Bartelink, Roger A. M. J. Damoiseaux

**Affiliations:** 1 Julius Center for Health Sciences and Primary Care, University Medical Center Utrecht, Utrecht University, Universiteitsweg 100, 3584 CX Utrecht, The Netherlands; 2 Department of Public Health and Primary Care, Ghent University, Corneel Heymanslaan 10, B-9000 Ghent, Belgium

**Keywords:** Evidence-based medicine, General practice, Family medicine, Workplace-based learning, Video-stimulated elicitation interviews, Learning conversations

## Abstract

**Background:**

To be able to practice evidence-based medicine (EBM) when making decisions for individual patients, it is important to learn how to combine the best available evidence with the patient’s preferences and the physician’s clinical expertise. In general practice training, these skills can be learned at the workplace using learning conversations: meetings between the supervising general practitioner (GP) and GP trainee to discuss medical practice, selected topics or professional performance. This study aimed to give insight into the perceptions of GP trainees on their EBM learning processes during learning conversations.

**Methods:**

We held semi-structured video-stimulated elicitation interviews (*n* = 22) with GP trainees affiliated to GP training institutes in the Netherlands and Belgium. GP trainees were shown fragments of their learning conversations, enabling reflection during the interview. Taking an inductive approach, interview recordings were transcribed verbatim and analysed with NVivo software.

**Results:**

GP trainees perceived learning conversations as useful for learning and discussing EBM. Multiple EBM learning activities were identified, such as discussing evidence together, relating evidence to cases in daily practice and discussing the supervisor’s experience and the specific local context in the light of what the evidence recommends. However, for learning to occur, trainees need and expect specific behaviour, both from their supervisors and themselves. Supervisors should supply well-substantiated answers that are applicable in practice and give the trainee confirmation. In turn, the trainee needs to prepare well in order to ask focused, in-depth questions. A safe space allowing equal and open discussion between trainee and supervisor is perceived as an essential context for optimal EBM learning.

**Conclusions:**

Our findings show that trainees find learning conversations useful for EBM learning in general practice. To bring EBM learning to its full potential, attention should be paid to optimising the behavioural and contextual factors found relevant to enhancing EBM learning.

## Background

Teaching and learning evidence-based medicine (EBM) in general practice, defined as combining current best evidence with the general practitioner’s (GP) own clinical expertise and the patient’s preferences, is important but complicated [1, 2]. According to the Sicily Statement, EBM involves five steps: ask a question, search for evidence, appraise that evidence for validity and clinical importance, apply the appraised evidence to practice and evaluate the result [3]. To provide best care for individual patients, clinicians and scientists advocate focussing on the last two steps of EBM [2, 4–6]. However, a recent review by Albarqouni (2018) showed that most educational interventions deal mainly with the first three steps [7]. Other reviews on EBM learning confirm these findings: although most educational programmes seek collaboration with clinical practice, their efficacy is unclear and incorporating all five steps is hard to achieve in the workplace [8–13]. To improve workplace-based teaching and EBM in general practice, it is important to look closely at how current teaching and learning takes place.

GP trainees learn by working together with GPs [14, 15]. Collaborative workplace-based learning happens in several ways, including deliberative learning. Deliberative learning occurs when time is set aside for learning activities at the workplace, leading to informal but yet planned forms of learning [16, 17]. It can be seen as a focused effort to improve performance, by organising moments of critical reflection individually or together with other health care professionals [18–20]. In the GP practice, learning conversations are commonly used as a form of deliberative learning. Learning conversations are regular meetings between GPs and trainees to discuss daily medical practice, selected topics or professional performance [21, 22]. These learning conversations, in which trainees actively nominate the topics or questions, are not often described in the literature. Performance assessment in the workplace often involves observation, evaluation and feedback, unilaterally from the supervisor’s point-of-view [23, 24]. However, recent work by Tavares and colleagues shows that it is possible to combine unilateral feedback with bidirectional processes such as debriefing and discussion, leading to learning conversations in daily practice [22]. We argue that a learning conversation might be a good EBM learning opportunity. For instance, GP trainees could learn EBM through reflective discussions with their supervisor about medical topics.

Not much is known about how learning conversations teach EBM learning. Applying EBM in daily clinical practice is often implicit and rarely a visible process, which might hamper any useful evaluation of EBM considerations [25, 26]. While EBM intends to help physicians make decisions by deliberatively reviewing all the information needed, the literature on the concept of mindlines shows that explicitly weighing all available knowledge and information can not always be achieved. In clinical reasoning and decision-making physicians seem to rely on internalised, collectively reinforced tacit knowledge that might be evidence-based but is hard to elucidate after the decision is made [27–29]. Using tacit knowledge may also hamper insightful discussions between supervisor and trainee, since supervisors and trainees might not be able to reflect or elucidate on EBM decision-making.

Since learning can be seen as a social and subjective process in which each learner constructs meaning, it is important to investigate the way that learners, in this case GP trainees, treat the evidence in learning conversations [30]. Trainees may perceive aspects that facilitate optimal EBM learning processes during learning conversations and other aspects which do not. This study sought to gain insight into the perceptions of GP trainees on EBM learning processes during learning conversations with the aim of obtaining a better understanding of workplace-based EBM learning and developing recommendations on how to optimise learning conversations.

## Method

### Study setting

This study was conducted in GP practices in the Netherlands and in Flanders, Belgium. General practice specialty training in the Netherlands and Flanders is comparable as in both countries, postgraduate medical training involves the trainee working alongside a GP for 2 years. Dutch trainees stay 1 year in a practice, while Belgian trainees can choose to stay with the same GP for both years. Formal education in both countries takes place at the training institute in small group classes; EBM training is a common aspect of these classes. Supervisors receive formal training (including EBM) in teach-the-teacher sessions. In both countries, supervisor and trainee hold regular workplace-based learning conversations during which GP trainees are expected to be responsible for their own learning process.

### Study design and participant recruitment

Using video-stimulated elicitation interviews (VSI) we conducted a qualitative multicentre study of 22 GP trainees affiliated with GP training institutes in Antwerp and Ghent (Belgium) or Utrecht (the Netherlands). Since we wanted to film active learning conversations between trainees and supervisors, we recruited established pairs of GP supervisors and GP trainees. We approached potential participants between September 2016 and April 2017, distributing flyers and giving promotional speeches at the training institutes and giving information on the study on a website. In Flanders, we used purposeful sampling to maximise variation [31]. Since recruitment in the Netherlands was more complicated due to unknown reasons we had to switch to convenience sampling here. After recruitment, participants filled out a short questionnaire on baseline characteristics (Table 1).

**Table 1:** Characteristics of participants

	GP trainees (*n* = 22)
Female	17 ^a^
Age (average in years (range))	28 (25–35)
PhD trajectory (finished or ongoing)	2
Trainee in first year of training	13
Trainee in last year of training	9
Experience of supervisor as GP (average in years (range))	23 (12–38)
Supervising experience of supervisor (average in years (range))	10 (1.5–25)
Duration of collaboration between supervisor and trainee (average in months, collected when starting the video-recordings (range))	8 (3–18)
Practice type	
Solo	2
Duo	9
Health centre	11
Training institute	
Utrecht	9
Antwerp	3
Ghent	10

^a^*: Results are numbers, unless stated otherwise*

### Data collection

Data collection took place between November 2016 and August 2017. Trainees were asked to video-record three regular learning conversations during their daily practice. Since learning conversations are a regular part of their workplace-based training, trainees and supervisors are accustomed to carry out these conversations, during which they together reflect on topics that occurred during daily practice or are relevant for daily practice. During their regular training at the institute, no specific instructions are given on how to carry out such learning conversations. For the collection of recordings for this study, we gave a few additional instructions. We asked the pairs to make video recordings of dialogues on a medical topic or question, since we expected that comments on personal development or communication skills, which participants might mention as well, would contain less useful EBM-related material. The three learning conversations were recorded over 4 months, a period long enough to take into account the development of the relationship between GP and GP trainee.

Afterwards, the first author (LW) selected two video fragments per trainee, not from the same recording. Fragments were considered suitable when the trainee asked the supervisor a medical question that led to a discussion between them. During the semi-structured interviews, held approximately within 2 weeks of the last video recording, trainees were first asked to talk in general on how they spoke about EBM with their supervisors and which aspects of EBM they found important in such conversations. Subsequently, the participants were shown the selected video fragments to deepen the interview and encourage reflection on these real situations. Socially desirable answers were minimised using this video-stimulated interviewing (VSI) technique [32–35]. Interviews followed the topic guide developed and iteratively revised by the research team. They lasted approximately 45 to 60 min and took place in private rooms at the GP practices.

### Analysis

All interviews were audio-recorded, transcribed verbatim and analysed using NVivo 11 software. An inductive approach was chosen to analyse the interviews [36]. To obtain a better view on the data and enhance reflexivity, the first two interviews were analysed separately by five researchers (LW, IvdW, EdG, MLB and KVR). The outcomes and different views were discussed and a provisory code tree was formed by identifying the main interview categories. Then three researchers (LW, IvdW and KVR) began coding interviews in rotating pairs, discussing the coding until consensus was reached. The last 12 interviews were coded individually by the same three researchers. Each individually coded interview was discussed by the rotating pairs and any queries were discussed at meetings of the full research team. After analysing all 22 interviews, we concluded that no new themes had emerged in the final interviews and that saturation was reached. Subsequently, using axial coding, we formed categories to present an overview of GP trainees’ perceptions of the EBM learning processes using learning conversations. We analysed the trainees’ general comments on their EBM learning process during a learning conversation, as well as their reflections on being confronted with the video of their own real-life learning conversations.

## Results

Thirteen Flemish and nine Dutch trainees were selected to participate. The group was heterogeneous: participants differed in their stage of training, practice type, their supervisor’s experience and the duration of collaboration with their supervisor (Table 1).

Interview analysis reveals that trainees perceived learning conversations as useful moments to learn and discuss EBM. Multiple EBM learning activities could be identified in the learning conversations. However, for learning to occur, trainees need and expect specific behaviour from their supervisor and themselves (Tables 2 and 3; see below).

**Table 2:** EBM learning activities that GPs trainees perceive as useful during learning conversations

*Useful EBM learning activities during learning conversations*	
• Reading evidence prior to the conversation and discussing it together • Looking up cases to illustrate the prepared evidence • Looking up evidence on the spot to answer a clinical question • Brainstorming and mutual discussion, applying the supervisor’s experience and advice gleaned from the evidence

**Table 3:** What GP trainees need and expect from learning conversations to enhance EBM learning

*Supervisor’s behaviour*	*Trainee’s behaviour*
• Substantiate answers with explicit argumentation • Give to-the-point answers that can be applied in practice • Give confirmation and reassurance • Ask counter questions • Point out new viewpoints • Elaborate on own approach or experience • Discuss patient’s preferences	• Ask focused questions • Keep on questioning if an answer is unclear • Prepare for the learning conversation in advance • Look up additional evidence afterwards to fill knowledge gaps • Try out and evaluate the new knowledge in daily practice
*In a context of:*	
• Equal, open and safe discussion between trainee and supervisor	

### EBM learning activities

Various EBM learning activities occurred during the conversations (Table 2). To acquire more knowledge, some pairs prepared for the learning conversation by reading evidence or guidelines in advance. During the conversation, trainee and supervisor discussed newly acquired knowledge and their views on what they had read, allowing for collaborative learning.

**Trainee 16**

*Interviewer: “Do you discuss such an article during the conversation, or how do you handle that evidence?”*

*Trainee: “Yes, we discuss that during […*]. *We often know beforehand what we’re going to read: this guideline, that article. We’ll email them in advance and then we’ll discuss what we’ve noticed, what we’ve learned for ourselves about something and how we’re going to apply it in this case. Because usually we’ll both tell each other, oh we didn’t know that, or that’s special.”*

Trainees stated that as they gained familiarity with the guidelines, the focus of the learning conversation shifted from purely acquiring guideline-based knowledge to trying to anticipate how they would put this knowledge into practice. We identified several ways of learning how to put evidence-based knowledge into practice. To begin with, trainees suggest cases from daily practice, using such examples in two ways. First, referring to exemplary cases helps trainees interpret the general advice given in the guidelines. Trainees noted that looking at different cases and discussing why their more experienced supervisor took a certain decision helped them interpret knowledge from the evidence.

**Trainee 11**

*“For example, hypertension. Recently I looked up the guideline again. I had my own cases and my colleagues had cases too so I reviewed [my colleagues’ cases] to check if I’d done anything differently, based on the guideline, or [to ask] why did you do it like that? So that’s actually very interesting.”*

Secondly, the pairs used exemplary cases as a starting point in the search for evidence. During the learning conversation, they looked for evidence when the trainee encountered a problem or dilemma in daily practice. They sought explicit answers to a clinical question, searching in easily accessible guidelines and websites where much of the evidence is pre-appraised; primary evidence was only very scarcely taken into account.

**Trainee 14**

*Interviewer: “Ok so you told me that you discuss the guideline together. How exactly do you do that? What kind of aspects do you discuss together?”*

*Trainee: “Gosh, it is goal-oriented: for example looking up [information] about medication and which medicine [to prescribe]. Yes, actually it’s usually case-oriented, so we’ll we look up how to do it for that patient or, yes, we’ll read through that together and then if there are things in it that, ah, we didn’t know, then we’d say so.”*

Another EBM learning activity trainees use in learning conversations is brainstorming and discussing with their supervisor. Brainstorming mainly concerns the interpretation of guidelines. For instance, trainees state that they struggle with specific recommendations for cut-off points or the practical interpretation of advice. Brainstorming starts when trainees bring their evidence-based knowledge to the table, whereupon supervisors interpret this information using their experience. Incorporating the local context is often useful in guiding the trainee’s interpretation.

**Trainee 3**

*“Yes, look, what they’re trying to do in the guidelines is […*] *set the cut-off values. [For] longer than a week, you do a faecal culture. Yes, do you do that after six days, or do you not do it after six days but after eight or seven days? You know, of course, they’ve chosen the cut-off values [for] the NHG standard. But in a learning conversation I’m trying to find out when I actually have to do something, and when not. [...] The standard isn’t always exactly clear on the cut-off value, I’ll say. I think that in a learning conversation you’re looking for those cut-off values for yourself, to get a grip on them, and you do that by sparring [with your supervisor] because then you get a reciprocal conversation.”*

### The supervisor’s behaviour

To facilitate optimal EBM learning, trainees need and expect specific behaviour from their supervisors (Table 3). First of all, supervisors should give well-substantiated, explicit answers to the trainee’s questions. A well-substantiated answer is not necessarily based on evidence. It can also be based on experience, on logical, pathophysiological reasoning or on patient- or context-related factors, as long as the answer is explicit and enables the trainee to reflect on the considerations that are used. The trainee needs to be able to decide whether they will use the argumentation in their own daily practice afterwards.

**Trainee 17**

*“In this situation, I take it on because he’s not forcing me, like [telling me] you have to do it like this because it’s really recommended that we do it that way. He frames it as look, I do it for this reason [but] I can still do what I want with it. He doesn’t force it on me. He does explain his viewpoint and why he does it.”*

Besides well-substantiated, the supervisor’s answers need to be to the point, practical and applicable in daily clinical practice to be useful for trainees. Trainees want straightforward answers to direct them in difficult cases, possibly due to time constraints or for convenience, since the learning conversation has a practical function in daily clinical practice: it gives trainees an easy way to gain information when time is running short and when they need clear answer on how to proceed in specific cases.

**Trainee 20**

*“Sometimes just from – okay these are the patients I definitely should discuss, the ones I want to see if I’ve treated correctly or the ones I know will call me back. Then I must have an answer to my question.”*

The need for direction can be related to something else the trainees mentioned needing: ‘confirmation’, which was perceived as an important element that could facilitate EBM learning. Confirmation that the supervisor, given their extensive experience, would do the same in the discussed situation is important for trainees to learn how to make clinical decisions in general practice.

**Trainee 5**

*Interviewer: “What does a learning conversation add for you?”*

*Trainee: “Well, if I’m wondering if something [I do] would be good medical treatment, then I check if it’s good and often they’ll say yes and then I feel reassured. So then it’s more to see that I don’t overlook anything and don’t make a mistake.”*

Trainees expected their supervisors to point out new points of view related to diverse aspects. Supervisors may point out new knowledge-based evidence, topics or guidelines which the trainee may not have been aware of, but it can also concern more case-related knowledge, such as discussing alternative diagnoses or noticing the trainee’s blind spots in clinical reasoning. Trainees perceived it as very useful when their supervisor did not answer their questions immediately but first makes them think for themselves. They valued it when the supervisor asked counter questions to clarify and specify their question or when the supervisor tested their line of reasoning.

**Trainee 22**

*Interviewer: “When do you feel that you’ve learned something from the conversation?”*

*Trainee: “Well, usually when XXX [supervisor] has a certain view of my case or she’s thinking in a certain direction. Sometimes XXX can switch your direction completely so that makes me think, ah yes I hadn’t looked at it that way or I should definitely think about it.”*

Finally, trainees felt that their supervisor’s help, in knowing and discussing the patient’s wishes, was important in enabling their EBM learning. This can concern discussing patients in general, such as how to deal with patients who ask for non-evidence-based medical treatment. Advice on how to deal with such patients is often based on their supervisor’s experience of working in a practice for a long time, which has led to deeper knowledge of ‘typical’ patients. However, learning conversations on the wishes of the patient are more often patient-specific: the supervisor and trainee discuss those patients with whom the supervisor often has a longer history. In this way, supervisors can help trainees to develop a broader outlook than just the medical problem of a specific patient by also taking context-related factors into account. This helps trainees make decisions which can at times judiciously diverge from the guidelines, in this way enhancing the application and evaluation of EBM.

**Trainee 7**

*Interviewer: Imagine that there is a case and you have doubts between treatment A and B. The diagnosis is quite clear to you but you don’t know how to proceed. How do you discuss such a question and how, what, when … do you learn from her [your supervisor’s] answer?*

*Trainee: “Yes, then XXX [supervisor] comes up with the arguments. Then she says, well in this situation, that patient lives like this or that, [and she gives me] a little more of her background knowledge and experience [...] of that particular patient in this situation. […]Yes. I really think that she’s good at pointing out patient-specific things and that’s also her argumentation.”*

Reflecting on the videos of their learning conversations, most trainees said they were content with their supervisor’s behaviour. However, supervisors seemed to find giving an explicit, well-substantiated answer the hardest thing to do in practice. Trainees felt that this might be partly their own ‘fault’, since they might be too easily satisfied if their supervisor gave what sounded like a convincing answer. This seemed to happen more often when trainees had no other starting points on what to do or how to proceed: in this case the non-substantiated answer of their supervisor gave them at least some direction.

**Trainee 12**

*Interviewer: “You tell me that, overall, when he [supervisor] explains something well using good argumentation, that you follow his advices. But what kind of argumentation do you find important to hear?”*

*Trainee: “So it’s based on [his] experience but frankly if I really have no idea and he’s quite convincing then I just say, well okay then.”*

### The trainee’s behaviour

Trainees acknowledged that their own behaviour and activities before, during and after learning conversations plays an important role in the effectiveness of the EBM learning process and that they too can be expected to behave in a certain way during the conversation (Table 3). They should be asking focused questions and keep on asking if and when the answer is not clear to them. The trainees mentioned that good preparation is essential for the learning conversation. Afterwards, they should make an effort to search the literature for solutions to unanswered questions and should try out and evaluate the themes and advice to form their own considerations. However, on seeing the video of their own behaviour during the learning conversations, trainees reflected that they often settle for unclear answers. On top of that, they said that the questions that they want to ask are often not focused enough due to lack of preparation. This meant they obtained less useful information or help from their supervisor.

**Trainee 14**

*Interviewer: “And, in the end, looking back at this, are you satisfied with the results of such a learning conversation?”*

*Trainee: “Then perhaps we should prepare even more. I should read the guideline in advance and write down my questions so that I can ask concrete questions that we can go over. Because now it’s chaotic at times [...] You lose sight of the overview. Yes. Then I’d also get more concrete answers.”*

Trainees felt that looking up additional evidence on their own to fill the knowledge gaps that the learning conversation revealed was an important way of learning EBM. However, trainees said that they did not often do this in practice, mostly due to a lack of time. Furthermore, trainees said that they usually did not try to gain further evidence-based knowledge after the learning conversation, since the supervisor’s suggestions and advice often gave them enough tools to proceed in daily practice. Thus additional information-gathering for the goal of EBM learning had no priority. Instead, the trainees did try out and evaluate their new knowledge during daily practice after the learning conversations.

**Trainee 6**

*Trainee: “I should really have got stuck into the literature, which I didn’t do by the way”*

*Interviewer: “No. But why do you think you didn’t?”*

*Trainee: “Actually, based on what I’d seen, I was satisfied. Yes, I thought it was just a local problem that should be treated locally. But if she came back with it I’d probably do [a literature search].”*

### Context: equal, open and safe discussion between trainee and supervisor

Trainees stated that a safe space is essential for optimal EBM learning, so that the trainee can dare to question the advice of the supervisor and conflicting views and evidence can be openly discussed. Trainees value it when the supervisor gives advice but does not force the trainee to follow this advice. A discussion on equal footing helps trainees to make their own considerations and judicious decisions. However, trainees said that their current learning conversations are not always equal discussions. When reflecting on their behaviour on the video-recordings, trainees said that they sometimes feel obstacles preventing them from asking in-depth questions and creating an open discussion. The obstacles can be related to their own behaviour, since they feel that they should look up the answers on their own and not ask their supervisor. On the other hand, trainees do not want their supervisor to feel tested or embarrassed when they sense that the supervisor does not know the answer.

**Trainee 11**

*Interviewer: “Is there a reason that you don’t ask such a follow-up question during the conversation?”*

*Trainee: “Yes, I’m not really thinking about asking [a follow-up question] because I think I could have looked it up for myself. Or yes [...] because I feel a bit like I’m testing my supervisor’s knowledge of the guidelines.”*

## Discussion

This study aimed to gain insight into the perceptions of GP trainees on EBM learning processes during workplace-based learning conversations. GP trainees perceive learning conversations as useful to learn and discuss EBM. Multiple EBM learning activities were identified, such as discussing evidence together, relating evidence to cases in daily practice and discussing the supervisor’s experience and the local context in the light of the evidence. However, for these learning activities to occur, trainees need and expect certain behaviour of their supervisor, such as giving well-substantiated answers that are practically applicable and that give the trainee confirmation. On the other hand, the trainee needs to prepare well in order to be able to ask specific, in-depth questions. Furthermore, only in a context of open, interactive discussion between trainee and supervisor, unfolding all EBM learning processes is possible.

### The role of tacit knowledge

We assumed that using tacit knowledge and mindlines would hinder optimal EBM learning because it would make supervisors and trainees unable to reflect or elucidate on EBM decision-making during their learning conversations. Our results show that supervisors did not always give well-substantiated answers based on argumentation to their trainees. The concept of mindlines might play a role here, making supervisors unable to explicitly substantiate their answers or elucidate the reasons for certain advice. Theories on the stages of adult learning show that experts, such as supervisors, rely more on tacit knowledge and intuitive, non-analytical decision-making, while novice learners depend more on analytical reasoning [37–39]. However, to improve performance in clinical practice, both explicit and implicit reasoning are important. As previous research suggests, gaining extensive experience through deliberate practice is important to obtain non-analytical, implicit ways of reasoning [39]. Learning conversations can facilitate deliberate practice, where trainees have the space to reflect on their actions and supervisors are able to give constructive underpinning to their advice, which trainees can take back to practice. However, our results show that providing exact elucidation of substantiations may be difficult and impossible at times due to the nature of mindlines and implicit knowledge. To solve this, supervisors should be made aware of the importance of elucidating their exact reasoning, and encouraged to make as much of their reasoning explicit as possible. Furthermore, trainees should be encouraged to ask in-depth questions and ask follow-up questions if when the answer is still unclear to them. This could help turn the supervisor’s tacit knowledge into a discussable topic during the learning conversation.

### Threshold concepts

Our results showed that trainees consider having a fruitful discussion and brainstorming with their supervisor useful EBM learning activities, but they also want straightforward, to-the-point answers that can be applied in daily practice. This apparent contradiction could be explained by looking at learning to apply EBM as a threshold concept. In 2003, Meyer and Land first described and developed threshold concepts in higher education. They can be defined as core ideas, essential to the mastery of a specific field, that need to be grasped [40]. Grasping the core idea leads to an ‘aha’ moment through integration of different learning elements, and marks an irreversible transformational shift in the identity of the learner. Learning and dealing with threshold concepts can often be troublesome: learners want to apply certain difficult skills, but fear making mistakes or that their knowledge is incomplete [41].

Looking at the skill of applying EBM, previous research states that dealing with the nature of evidence or uncertainty can be seen as threshold concepts that need to be mastered in medical education, particularly in general practice [42–44]. Specifically, Sokol and colleagues showed that formal classes on evidence-based medicine can lead to transformative learning when the learner masters such threshold concepts as ‘uncertainty is an aspect of medical decisions, but steps can be taken to proceed confidently’. [45]

Our results show similar effects, since in the learning conversations we observed trainees trying to master threshold concepts related to using EBM in daily clinical practice. Troublesome signs were apparent, since trainees described their need for help if they felt unsure or were unable to apply evidence in a straightforward manner. This explains the trainees’ paradox: on the one hand wanting to discuss EBM and be supported in forming their own considerations, but on the other hand wanting direction and confirmation from their supervisor. Trainees use learning conversations as educational moments that help them master these concepts.

We would like to suggest that supervisors should help trainees master EBM-related threshold concepts even more during learning conversations. For instance, they could encourage trainees to let go of the idea that there are absolute answers in medicine and accept the fact that decision-making in general practice comes with uncertainty. EBM is not just only about searching and appraising evidence. It must be applied in practice, in combination with the patient’s preferences and the clinician’s own clinical expertise.

### Equal, safe and interactive discussion

An important outcome of our research was that optimal EBM learning depends on creating a safe, equal and interactive space in which conflicting views and new information can be openly shared and discussed. The literature on professional learning conversations finds this an important prerequisite as well. Earl and Timperley state that *“the basis of learning conversations is the mutual understanding of each contributor’s claims and the values, together with the reasoning and data on which they are based”* [46]. Other research also emphasises the need for open, safe discussion with learner participation and an educator who actively engages the learner in interactive discussion as both factors are important for giving feedback or debriefing [22, 47, 48]. However, the role of this open environment has not been clearly described specifically for EBM learning in workplace-based learning conversations. Research does acknowledge the important role of a safe environment for EBM learning in general, and has even led to an instrument that characterises the EBM learning environment [49]. Another study with surgical residents, on barriers to the use of EBM, also describes the residents’ fear of confronting supervisors with new evidence, implicitly saying that their current practice is outdated and possibly embarrassing them [50]. The review by Van Dijk et al. on barriers for trainees to practice EBM also mentions the role of the learning climate as important to the EBM behaviour of trainees [51]. Specifically, a climate with hierarchical dependence hinders EBM learning and expressing information needs, making *‘safe communication and shared learning across career stages perceived as the most prominent facilitator for EBM’*. [52] This is in line with our findings in this study, as trainees stated that they find an equal and safe environment is not only important for EBM learning and practice in general, but it is also important for EBM learning during learning conversations in particular.

### Strengths and limitations

#### Strengths

To our knowledge, this study is the first to look specifically at how trainees perceive workplace-based EBM learning during learning conversations in general practice. By shedding light on how trainees experience these conversations and which EBM learning activities they execute, this study can start filling the current gap on how best to teach and learn the full spectrum of EBM in the workplace.

Our VSI methodology is a strength because the video recordings not only allowed us to shed light on how GP trainees handle and discuss evidence, it gave the trainees the opportunity to reflect on daily practice. This technique can identify more specific points of improvement.

Finally, when looking at learning and knowledge as social constructs, these interviews are the right approach to unfolding current learning processes [30]. Learning is a process of interaction and can best be examined by asking learners themselves what they have learned. Moreover, it can reveal implicit learning processes that might occur during the conversations but are not clearly visible, or elucidate the learning that occurs before or after the actual encounter that the learning conversation has initiated.

### Limitations

The participant sampling method may have influenced our results, since we had to switch to convenience sampling in the Netherlands. In theory, this might have led to less rich results due to reduced variation in the characteristics of participants, or selecting only trainees with a pronounced interest in EBM. However, when we look at the characteristics and composition of the Belgian and Dutch groups of trainees, we do not expect the sampling method to have had any significant impact on our results, since variation within both national groups was similar.

Secondly, our videos of learning conversations could have shown socially desirable EBM behaviour, since the participants were aware of the EBM-oriented goal of this study. To avoid this problem, however, we asked participants to record three learning conversations in full length (20–60 min), since research shows that awareness of being filmed fades when the recording continues for a longer period of time [53, 54]. Furthermore, we used the video fragments only as a starting point to talk about the way participants perceived EBM learning during the learning conversations. The fragments were used solely to deepen the interview and enable reflection.

## Conclusion and recommendations

This study demonstrates that learning conversations can be a useful way to improve and enhance EBM learning in general practice. To bring EBM learning in these conversations to its full potential, attention should be given to optimising the described behavioural and contextual factors that help EBM learning activities to take place. This includes encouraging trainees to prepare for their learning conversations thoroughly, so that they can ask specific, in-depth questions that stimulate supervisors to substantiate their answers and advice. Finally, creating an equal, safe and open space that allows room for discussion and brainstorming can improve EBM learning activities.

## Data Availability

The datasets used and/or analysed during the current study are available from the corresponding author on reasonable request.

## Abbreviations

EBM: Evidence-based medicine
GP: General practitioner / general practice
VSI: Video-stimulated elicitation interview
NVMO: *Nederlandse Vereniging voor Medisch Onderwijs /* Dutch Society of Medical Education
NHG: *Nederlands Huisartsen Genootschap* / Dutch College of General Practitioners
LW: Lisanne Welink
EdG: Esther de Groot
PP: Peter Pype
KVR: Kaatje Van Roy
IvdW: Iris van den Wittenboer
MLB: Marie-Louise Bartelink
RD: Roger Damoiseaux
